# Effects of Chronic Social Stress and Maternal Intranasal Oxytocin and Vasopressin on Offspring Interferon-γ and Behavior

**DOI:** 10.3389/fendo.2016.00155

**Published:** 2016-12-14

**Authors:** Christopher A. Murgatroyd, Alexandria Hicks-Nelson, Alexandria Fink, Gillian Beamer, Kursat Gurel, Fawzy Elnady, Florent Pittet, Benjamin C. Nephew

**Affiliations:** ^1^Manchester Metropolitan University, Centre for Healthcare Science Research, Manchester, UK; ^2^Department of Biomedical Sciences, Cummings School of Veterinary Medicine, Tufts University, North Grafton, MA, USA; ^3^Department of Biology, Stonehill College, Easton, MA, USA; ^4^Department of Infectious Disease and Global Health, Cummings School of Veterinary Medicine, Tufts University, North Grafton, MA, USA; ^5^Department of Anatomy and Embryology, Faculty of Veterinary Medicine, Cairo University, Cairo, Egypt

**Keywords:** social stress, depression, depression and anxiety disorders, interferon-γ, oxytocin, vasopressin, social behavior, inflammation

## Abstract

Recent studies support the hypothesis that the adverse effects of early-life adversity and transgenerational stress on neural plasticity and behavior are mediated by inflammation. The objective of the present study was to investigate the immune and behavioral programing effects of intranasal (IN) vasopressin (AVP) and oxytocin (OXT) treatment of chronic social stress (CSS)-exposed F1 dams on F2 juvenile female offspring. It was hypothesized that maternal AVP and OXT treatment would have preventative effects on social stress-induced deficits in offspring anxiety and social behavior and that these effects would be associated with changes in interferon-γ (IFNγ). Control and CSS-exposed F1 dams were administered IN saline, AVP, or OXT during lactation and the F2 juvenile female offspring were assessed for basal plasma IFNγ and perseverative, anxiety, and social behavior. CSS F2 female juvenile offspring had elevated IFNγ levels and exhibited increased repetitive/perseverative and anxiety behaviors and deficits in social behavior. These effects were modulated by AVP and OXT in a context- and behavior-dependent manner, with OXT exhibiting preventative effects on repetitive and anxiety behaviors and AVP possessing preventative effects on social behavior deficits and anxiety. Basal IFNγ levels were elevated in the F2 offspring of OXT-treated F1 dams, but IFNγ was not correlated with the behavioral effects. These results support the hypothesis that maternal AVP and OXT treatment have context- and behavior-specific effects on peripheral IFNγ levels and perseverative, anxiety, and social behaviors in the female offspring of early-life social stress-exposed dams. Both maternal AVP and OXT are effective at preventing social stress-induced increases in self-directed measures of anxiety, and AVP is particularly effective at preventing impairments in overall social contact. OXT is specifically effective at preventing repetitive/perseverative behaviors, yet is ineffective at preventing deficits in overall social behavior.

## Introduction

Alterations in maternal care can mediate the developmental consequences of early-life experiences. Early maternal–infant interactions serve as a potential source of information concerning the environment to which offspring will need to adapt. For example, levels of maternal care can profoundly influence stress physiology in the infant and their developmental trajectory (1). Changes in infant glucocorticoid responses can be altered by the mother’s behavior, eliciting lasting alterations in glucocorticoid responsiveness, and related behavioral changes (2). Further studies have shown that alterations in maternal care can be non-genomically inherited. Wild-type offspring born to a mother mutant for the Peg3þ/(paternally expressed gene 3) gene, which shows impairments in various aspects of maternal behavior, exhibited a reduction in their own ability to retrieve pups to a nest in a retrieval test (3). We have also demonstrated that chronic social stress (CSS) can alter levels of maternal care behavior and associated neuroendocrine changes in offspring (4, 5).

However, the mother’s influence extends beyond classic neuroendocrine stress response systems. Early-life stress and maternal care is able to activate not only neuroendocrine systems but also the innate immune system, which effects behavioral responsiveness (6, 7). The presence of the mother effectively suppresses the behavioral consequences of innate immune activation (8). Recent studies have supported the hypothesis that the adverse effects of early-life adversity on neural plasticity and behavior are mediated by inflammation (9). For example, mice deficient in adaptive immunity exhibit social deficits and hyper-connectivity of fronto-cortical brain regions, which are mediated by interferon-γ (IFNγ) (10), a key regulator of immune responses (11). These findings introduce the possibility that long-term adaptive behavioral change can be mediated by the mother’s influence on immune-related activity of her pups. This further raises the question on the role of maternal neuroendocrine factors in infant immune activity. Concerning the present study, it is known that vasopressin (AVP) and oxytocin (OXT) stimulate T-helper cells to produce IFNγ (12), and OXT treatment suppresses TNFα-production in LPS-stimulated microglial cells and a decrease in microglial activation *in vivo* in rats (13).

**Figure 1:**
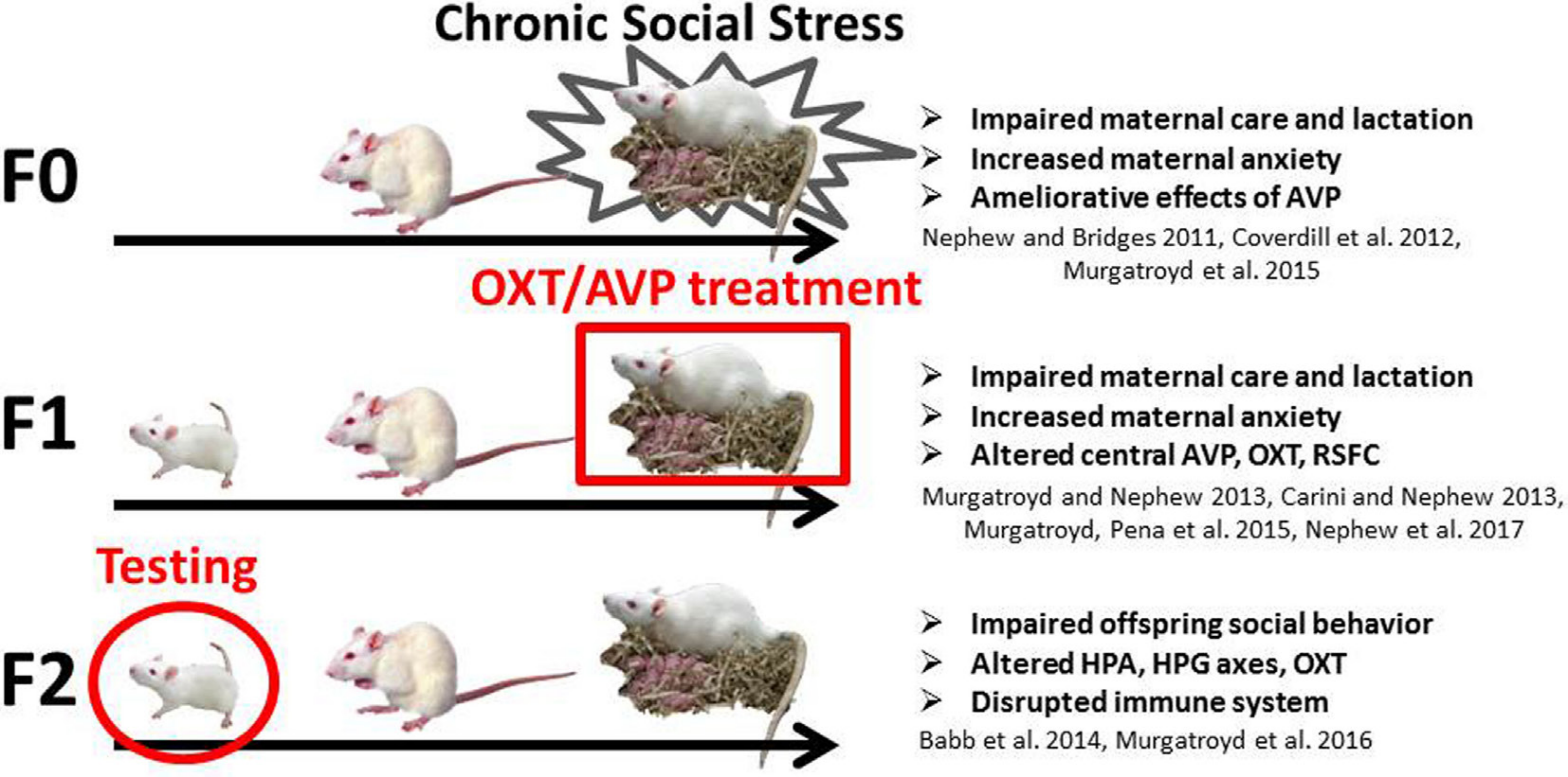
**Diagram of the chronic social stress model**. The current study focuses on the F2 juvenile female offspring (circled) of intranasal vasopressin- and oxytocin-treated F1 dams.

The CSS model of postpartum depression and anxiety (see Figure 1) depresses maternal care, impairs lactation, and increases maternal anxiety in F0 rat dams exposed to chronic male intruder stress during days 2–16 of lactation (14–16) and has similar effects in F1 and F2 dams (4, 17, 18). Juvenile and adult F2 offspring of F0 CSS dams exhibit deficits in social behavior (5). For the F1 and F2 offspring of stressed dams, the effects of CSS on social behavior may be mediated by early-life exposure to depressed F0 maternal care and/or the male intruder stressor (F1 offspring) or depressed F1 maternal care only (F2 offspring). At the neuroendocrine level, CSS F0 dams have decreased OXT gene expression in the MeA, and CSS F1 dams also have lower MeA OXT, lower AVP in the MeA and PVN, and higher OXTR gene expression in CeA (18). CSS F2 juvenile females have higher basal serum OXT levels (5) though basal OXT levels in the CSS F1 dams do not differ (4), suggesting the transgenerational accumulation of the effects of social stress. Furthermore, female F2 adult offspring of CSS dams exhibit decreases in the immune factors alpha 1 acid glycoprotein (α1AGP) and intercellular adhesion molecule 1, and α1AGP levels are correlated with allogrooming during a social behavior test (7) supporting the role of immune factors in behavioral programing by maternal care. Chronic AVP infusion into the lateral ventricles improves maternal care in CSS-exposed F0 dams (19), but treatment of the F1 or F2 generations has not been attempted.

Both animal and clinical studies have indicated that intranasal (IN) OXT has potent effects on parental behavior (20, 21). Acute OXT studies in animals stimulated clinical studies using acute and chronic IN dosing, despite a lack of preclinical data with chronic dosing. Recent rodent studies report that chronic IN OXT may have adverse long-term effects on social behavior (22), and while IN OXT is being tested as a potential treatment for postpartum depression and anxiety with mixed results (23, 24), the effects of maternal OXT, or closely related AVP, on offspring behavior and physiology have not been thoroughly studied. The objective of the present study was to investigate the immune and behavioral programing effects of IN AVP and OXT treatment of CSS-exposed F1 dams on F2 juvenile female offspring. It was hypothesized that both AVP and OXT would have preventative effects on social stress-induced deficits in social behavior and increased anxiety and that these effects would be associated with changes in TNFα and/or IFNγ in juvenile F2 females. It was predicted that CSS would decrease F2 IFNγ and increase TNFα, and F1 OXT and AVP treatment would prevent these changes, resulting in similar immune and behavioral levels in controls and the F2 offspring of OXT- and AVP-treated controls.

## Materials and Methods

### Animals

Sprague-Dawley rats (Charles River Inc., Kingston, NY, USA) in this study were maintained in accordance with the guidelines of the Committee of the Care and Use of Laboratory Animals Resources, National Research Council, and the research protocol was approved by the Tufts Institutional Animal Care and Use Committee. “CSS dams” refers to the adult females exposed to CSS during lactation (F0), “CSS F1 dams” refers to the maternal adult female offspring of the CSS F0 dams, which were treated with chronic saline, AVP, or OXT, and “CSS F2 juveniles” refers to female juvenile F2 offspring, which are the focus of the present study. The five experimental groups consisted of control (CON), control + intranasal saline (CON SAL), CSS + intranasal saline (CSS SAL), CSS + intranasal AVP (CSS AVP), and CSS + intranasal OXT (CSS OXT). Open-field and social behavior were tested on days 37–38, the marble burying test was done on days 38–39, and all rats were euthanized on days 39–40 (the day following the marble burying test) between 0900 and 1100 hours.

### CSS Model: F0 Dams

The CSS dams were subjected to the CSS protocol from days 2 to 16 of lactation as reported (14, 15). This procedure consisted of placing a similarly sized (220–300 g) novel male intruder into a lactating female’s home cage for 1 h from days 2 to 16 of lactation. CON dams were not exposed to the CSS protocol. The pups were left in the cage during the novel male intruder presentation, and the CSS exposure results in reduced maternal care (pup grooming and nursing) and increased anxiety-related behavior and maternal aggression in F0 dams (14), creating an early-life stress of depressed maternal care and social conflict for the F1 generation.

### CSS Model: F1 Females

The CON and early-life CSS F1 females were the offspring of the F0 CON and CSS dams; the differences between the treatments of the CON and early-life CSS F1 females consisted of the exposure of the CSS F1 females to attenuated maternal care and conflict between their F0 mothers and the male intruders during age 2–16 days. The F1 CON and early-life CSS animals were treated identically after the age of 16 days. After weaning all F1 pups on day 23, the female offspring from the 12 CON and 12 CSS dams were housed in groups of four until 70 days of age, when 1–2 females from CON and CSS litters were mated with breeder males to obtain 12 CON and 18 CSS F1 females.

#### F1 Dam Intranasal Treatment Groups and Their F2 Offspring

F1 dams from a CSS background were randomly assigned to one of the three possible IN interventions: CSS SAL, CSS AVP, and CSS OXT, while F1 dams from a control background were randomly assigned to one of the two possible groups: CON and CON SAL. F1 CON dams were kept in a separate housing room because of the daily intrusion the other groups experienced for IN administrations. OXT and AVP (Sigma) or sterile saline alone were administered at a dosage of 0.8 IU/kg in 25 µl sterile saline based on previous studies (22). IN administrations occurred daily for 3 weeks of lactation. Rats were restrained using a flexible plastic cone called a DecapiCone (Braintree Scientific), which provided access to the nostrils and allowed for rapid and consistent administrations (20–30 s). IN fluids were administered using a 100 µl pipettor and rigid plastic non-puncturing pipette tips. Half doses were administered to each nostril.

Total F2 pup number and litter weights were recorded on the day of parturition, and litters were then culled to four females and four males. Other than the described IN treatments, the F2 groups of animals were treated identically throughout the study. The final F2 female juvenile sample sizes were seven for CON, six for CON SAL, eight for CSS SAL, eight for CSS AVP, and nine for CSS OXT. There were no treatment differences in F2 litter size or number or bodyweights at day 40 (all *p*’s > 0.1).

### Behavioral Tests

#### Open-Field and Social Behavior Testing

The experimental female rat was removed from the home cage and placed in a clean Plexiglas cage with black walls and a white floor (12″ × 20″ × 12″) for 5 min to allow for locomotor acclimation to the novel environment and video record open-field behavior. Videos were scored using Odlog (Macropod, Inc.). Open-field behaviors scored consisted of the durations and frequencies of moving along the edge (outer 2″), stationary along the edge, rearing along the edge, moving in the center, stationary in the center, rearing in the center, and self-grooming. At the end of the 5-min open-field test, one black wall was replaced with a clear barrier with a 0.5″ window 1″ from the floor. A same age novel rat from the same treatment group was in a same size cage on the other opposite side of the clear barrier. After 10 min, the clear barrier was removed to allow for direct social interaction for another 10 min. Social behaviors scored included rostral and caudal investigation, lateral contact, dorsal contact, allogrooming, self-grooming, locomotor activity, aggression, and total social contact (the sum of investigation, contact, and allogrooming).

#### Marble Burying Test

The marble burying test was conducted to assess repetitive and perseverative behavior (25). Juvenile female rats were placed in a clean cage with bedding where six marbles were evenly spaced on top of the bedding for 15 min. The number of marbles completely buried or at least 75% covered were counted at the end of 15 min.

### Measurement of IFNγ and TNFα

All experimental animals were euthanized within 3 min of entering the animal room between 0900 and 1100 hours, the day following social behavior testing, and trunk blood was collected for the analysis of TNFα and IFNγ. These were measured by individual rat ELISAs (R&D Systems, USA). Samples were run in duplicate in an individual assay to eliminate interassay variation, and intraassay variability was 4%.

### Statistical Analyses

Basal cytokine and behavior levels were analyzed with one-way ANOVA (on all five treatment groups and four groups with the two control groups combined) as well as *t*-tests of the combined control groups compared to the combined CSS groups to assess the overall effect of CSS (Table 1). These CSS-focused tests were followed by additional *t*-tests comparing individual treatment groups if there were significant treatment effects with ANOVA or the combined CON vs. combined CSS *t*-test. There were no differences between the CON and CON SAL groups with any of the variables, so these groups were combined to compare to individual CSS groups. All graphical results are presented as mean + SEM, significance was denoted as *p* ≤ 0.05, and *p* values refer to two-tailed tests unless noted. The use of one-tailed tests was justified for use with IFNγ based on previous findings of its importance in neuronal connectivity and social behavior (10) and recently published data on impaired functional connectivity in the CSS model (26). The use of one-tailed tests was justified for use with behavioral data by initial effects of CSS in the present study or previous reports of increased anxiety and decreased social behavior in the F2 generation of CSS model (5). Cohen’s *d* effect size tests were used to assess effect sizes, with 0.2–0.5 considered a small effect, 0.5–0.8 a medium sized effect, and values greater than 0.8 a large effect. Pearson correlations were used to test for significant IFNγ-behavior associations.

**Table 1:** ***t*-Test and Cohen’s *d* values of combined control groups vs. combined chronic social stress (CSS) group comparisons**.

Variable	Combined controls	Combined CSS	*p*-Value	Cohen’s *d*
IFNγ (ng/ml)	7.2 ± 2.9	17.9 ± 4.0	0.04	0.7
Marbles buried	2.9 ± 0.4	3.6 ± 0.2	0.05*	0.6
Self-grooming duration (s)	4.1 ± 1.5	11.5 ± 3.9	0.03*	0.9
Social contact duration (s)	50.3 ± 4.3	37.9 ± 4.1	0.03*	0.9
Allogrooming duration (s)	10.3 ± 4.0	3.7 ± 2.9	0.04	0.6

*Indicates one-tailed t-test.

## Results

### IFNγ and TNFα

**Figure 2:**
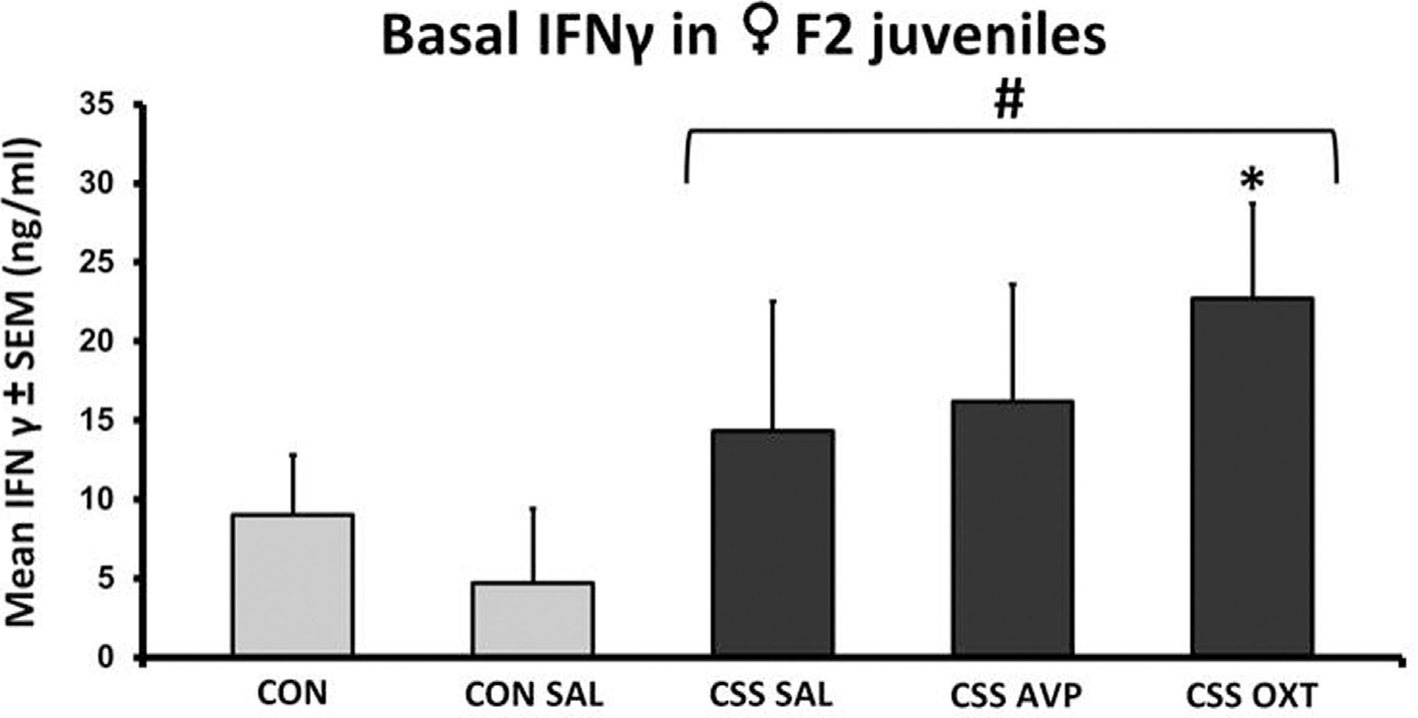
**Mean + SEM of basal IFNγ levels of chronic social stress (CSS) F2 juvenile female offspring of F1 control (CON) or CSS dams intranasally treated with saline, vasopressin, or oxytocin**. ^#^ indicates overall effect of CSS treatment, * indicates significant increase compared to combined CON and control + intranasal saline (CON SAL) groups and CON SAL alone (*p* < 0.05).

Basal plasma levels of TNFα were undetectable. There were no significant differences in IFNγ following one-way ANOVA (*F*_4,38_ = 1.0, *p* = 0.4, *F*_3,38_ = 1.3, *p* = 0.3, Figure 2). Basal plasma IFNγ levels were increased more than two-fold in combined CSS F2 offspring compared to combined CON F2 offspring (Table 1). This difference was driven by the CSS OXT group (22.7 ± 6.0 ng/ml, Figure 2), which had higher levels than the combined control groups (*t* = 2.5, *p* = 0.02, Cohen’s *d* = 1.1) and when compared only with the CON SAL group (one-tailed *t* = 2.0, *p* = 0.03, Cohen’s *d* = 0.3).

### Marble Burying

**Figure 3:**
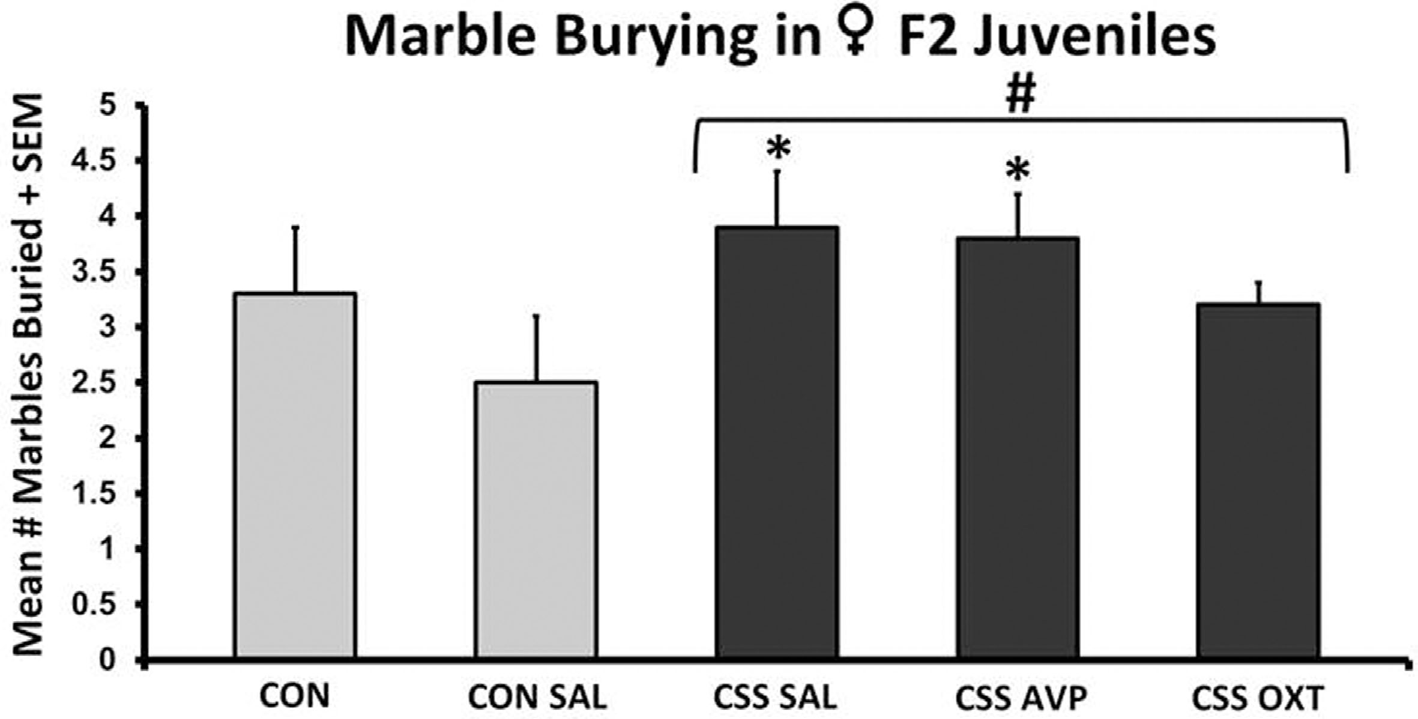
**Mean + SEM number of marbles buried by chronic social stress (CSS) F2 juvenile female offspring of F1 control or CSS dams intranasally treated with saline, vasopressin, or oxytocin**. ^#^ indicates overall effect of CSS treatment, * indicates significant increase compared to control + intranasal saline group (*p* < 0.05).

There were no significant differences in marble burying following one-way ANOVA (*F*_4,38_ = 1.5, *p* = 0.2, *F*_3,38_ = 1.5, *p* = 0.2, Figure 3). CSS-exposed F2 female juveniles buried more marbles compared to CON animals during the 15-min test (Table 1). When comparing individual treatment groups, both the CSS SAL (3.9 ± 0.5 s, one-tailed *t* = 1.8, *p* < 0.05, Cohen’s *d* = 1.0) and CSS AVP (3.8 ± 0.4 s, one-tailed *t* = 2.0, *p* = 0.03, Cohen’s *d* = 1.0) groups buried more marbles than the CON SAL group (2.5 ± 0.6 s, Figure 3). CSS F1 dam treatment with OXT decreased the number of marbles buried by CSS F2 female juveniles to a level similar to controls.

### Open-Field Behavior

**Figure 4:**
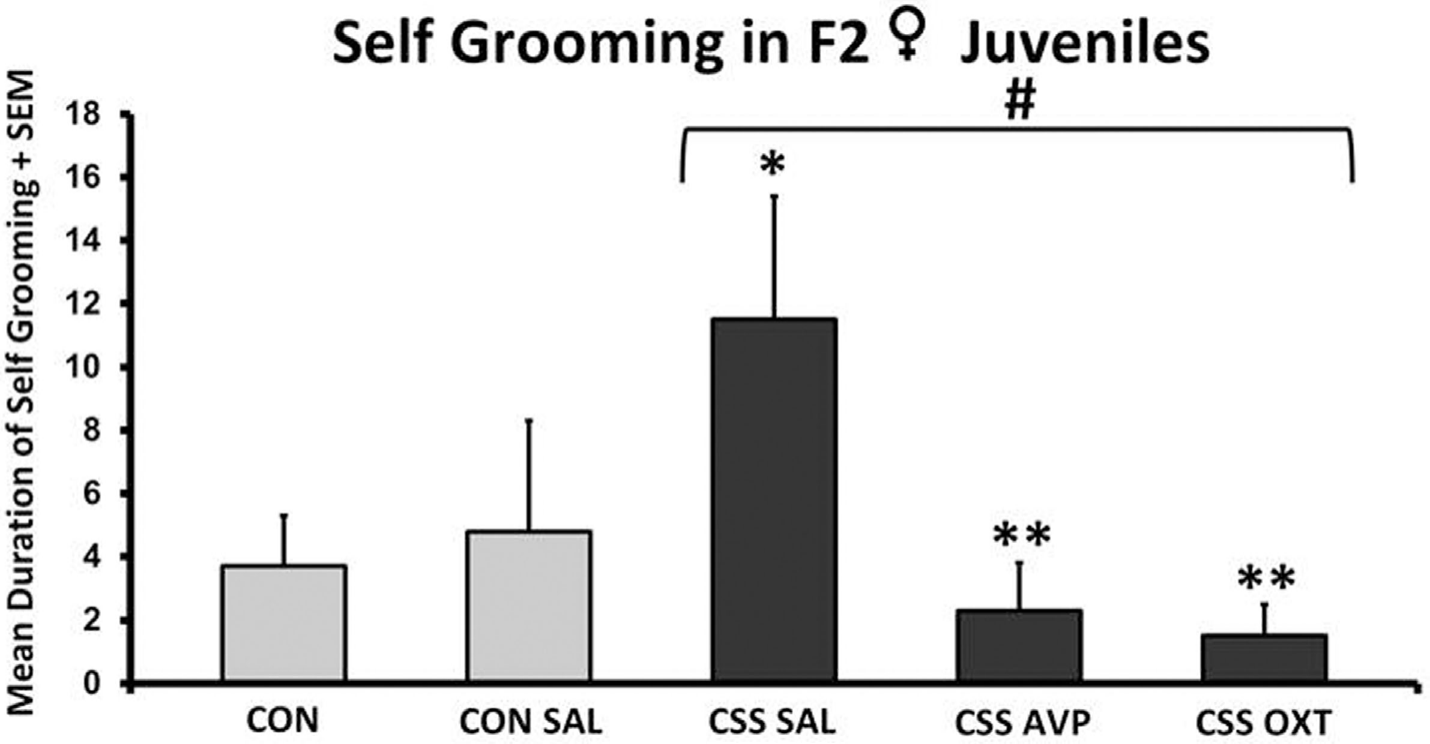
**Mean + SEM duration (seconds) of self-grooming of chronic social stress (CSS) F2 juvenile female offspring of F1 control (CON) or CSS dams intranasally treated with saline, vasopressin, or oxytocin**. ^#^ indicates overall effect of CSS treatment, * indicates significant increase compared to combined CON and control + intranasal saline groups, ** indicates significant decrease compared to CSS + intranasal saline group (*p* < 0.05).

There were no significant differences between CON and CSS-treated F2 female juvenile between any treatment groups in durations or frequencies of moving along the edge of the open field, stationary along the edge, rearing along the edge, and moving in the center of the open field (one-way ANOVA, all *p*’s > 0.3). Durations and frequencies for stationary and rearing in the center were too low for statistical comparison. There were no significant differences in self-grooming in the open field following one-way ANOVA with all five treatment groups (*F*_4,38_ = 2.6, *p* = 0.06), but there was a significant effect of treatment when the two control groups were combined (*F*_3,38_ = 3.5, *p* = 0.03, Figure 4). CSS SAL juveniles expressed an almost three-fold increase in mean duration of self-grooming during open-field testing compared to the combined control groups (Table 1). Mean self-grooming durations in both the CSS AVP (2.3 ± 1.5 s) and CSS OXT (1.5 ± 1.0 s) groups were lower than grooming in the CSS SAL group (both one-tailed *t* tests = 2.1, *p* = 0.03, Cohen’s *d* = 1.1, Figure 4), and similar to control values.

### Social Interaction

**Figure 5:**
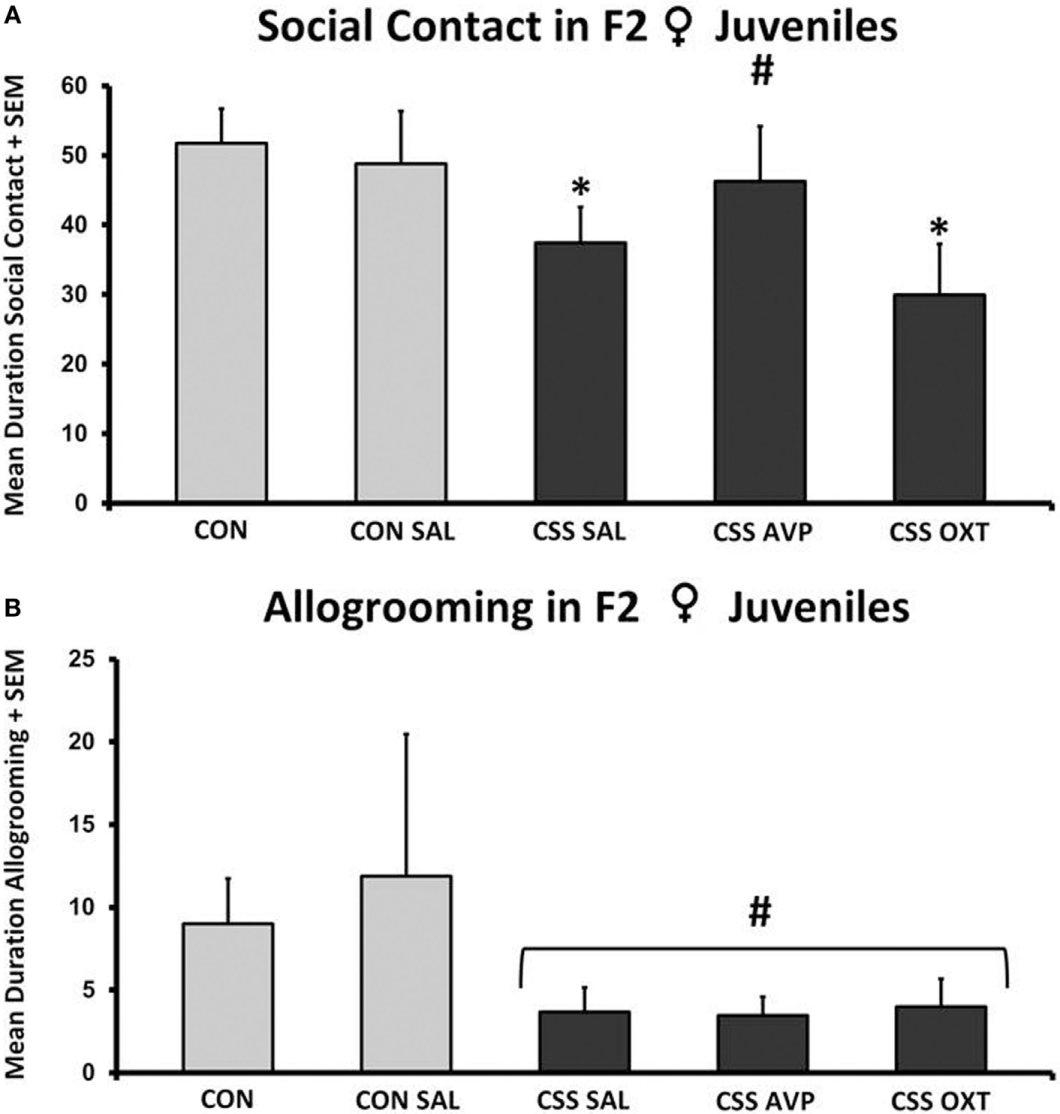
**(A)** Mean + SEM duration (seconds) of social contact of chronic social stress (CSS) F2 juvenile female offspring of F1 control (CON) or CSS dams intranasally treated with saline, vasopressin (AVP), or oxytocin (OXT). ^#^ indicates overall effect of CSS treatment, * indicates significant increase compared to combined CON and control + intranasal saline groups (*p* < 0.05). **(B)** Mean + SEM duration (seconds) of allogrooming of CSS F2 juvenile female offspring of F1 CON or CSS dams intranasally treated with saline, AVP, or OXT. ^#^ indicates overall effect of CSS treatment (*p* < 0.05).

There were no significant differences in social interaction following one-way ANOVA (*F*_4,38_ = 1.7, *p* = 0.2, *F*_3,38_ = 2.4, *p* = 0.09, Figure 5A). Combined juvenile female F2 CSS rats spent 25% less time displaying social behavior during the 10-min social interaction test compared to combined CON rats (Table 1). This overall difference was due to significant differences between the combined controls and the CSS SAL (37.4 ± 5.2 s, one-tailed *t* = 1.9, *p* = 0.04, Cohen’s *d* = 0.7) and CSS OXT (29.9 ± 7.3 s, *t* = 2.6, *p* = 0.02, Cohen’s *d* = 1.1, Figure 5A)-treated groups. Social contact duration in CSS F2 juvenile offspring of AVP-treated F1 dams was similar to control values.

There were no significant differences in allogrooming following one-way ANOVA (*F*_4,38_ = 1.2, *p* = 0.3, *F*_3,38_ = 1.5, *p* = 0.2, Figure 5B). When CON and CSS groups are combined, durations of allogrooming were decreased in CSS animals compared to controls (Table 1).

### IFNγ—Behavior Correlations

There were no significant correlations between basal peripheral IFNγ levels and durations of marble burying, self-grooming, social contact, or allogrooming (all *p*’s > 0.1).

## Discussion

Recent interest in the role of the immune system and IFNγ in behavior and the pathophysiology of stress-associated psychiatric disorders stimulated the present investigation of the effects of maternal IN AVP and OXT on programing changes in peripheral immune factors and behavior in juvenile offspring. Relevant behavioral models are needed to determine the mechanisms of these interactions in the context of early-life care and its impact on later disease. The current study reports increased peripheral IFNγ in the juvenile female offspring of social stressed dams treated with chronic IN OXT. CSS offspring also displayed increased perseverative and anxiety behavior, impaired social behavior, and behavior-specific responses to both maternal AVP and OXT treatment. The behavioral changes were not correlated with peripheral IFNγ levels, and it is postulated that maternal IFNγ indirectly mediates behavioral programing of offspring through neurodevelopmental changes (10). The data support the hypothesis that social stress and IN peptide administration in mothers alter peripheral immune measures and perseverative, anxiety, and social behavior in female offspring in a behavior- and peptide-specific manner.

### F1 Maternal OXT Drives Elevated Basal IFNγ in CSS F2 Offspring

Vasopressin and OXT have substantial immune functions and modulate the immune system during its development, homeostasis, and in response to injury and stress. AVP is emerging as a critical immunoregulatory peptide, capable of maintaining immune function. This is due to the ability of AVP and OXT to stimulate both the HPA axis and prolactin release (27). The genes for OXT and OXTR are expressed in the thymus (28) and monocytes and macrophages (29). OXT is the target of immunological cytokines (e.g., prostaglandin E2, IL2, and IL6) and prolactin, which can promote its secretion into the blood (30–33). Johnson et al. (34) demonstrated that AVP and OXT were able to replace the IL2 requirement for IFNγ production by T-helper cells from mouse spleen cultures. Importantly, this did not involve cell proliferation, suggesting that these neuropeptides possess cytokine activity supporting a relationship between neuroendocrine and immune systems (34). Further work demonstrated that AVP and OXT can replace IL2 for T cell mitogen induction by IFNγ in mouse spleen cells (12), and OXT significantly increases peripheral blood mononuclear cell blastic response to phytohemagglutinin (35). Based on these findings and the current data, it is hypothesized that AVP and OXT act as regulators of immune cells.

Elevated basal IFNγ in CSS F2 juvenile females was driven by the maternal OXT treatment, suggesting a specific effect of this neuropeptide on IFNγ and general immune functioning. The developmental importance of IFNγ (10) suggests that there may be substantial neuroanatomical changes in CSS F2 offspring. Robust changes in resting-state functional connectivity have been documented in studies of CSS F1 offspring (26), and detailed neuroanatomical and neuroimmunological investigation of the CSS F2 generation and similar populations would be valuable.

### F1 Maternal OXT Prevents Repetitive/Perseverative Behavior in CSS F2 Offspring

The overall increase in CSS F2 marble burying augments our previous report of social anxiety in this generation (5), supporting the hypothesis that CSS has transgenerational effects on both social anxiety and repetitive/perseverative behavior. In a natural setting, increased perseverative behavior could lead to decreased expression of critical behaviors such as foraging, sexual behaviors, and/or parental care. The CSS model is characterized by the increased expression of inappropriate behaviors, such as excessive nesting, locomotor activity, and unnecessary pup retrieval when maternal care is depressed in F0 and F1 dams. These behaviors have been referred to as maternal anxiety, but they may also reflect increased perseveration in stressed dams. A lack of a significant increase in marble burying in the CSS OXT group compared to significant increases in the CSS SAL and CSS AVP groups suggests that OXT may be specifically effective at preventing perseveration in the offspring of mothers exposed to early-life social stress. While AVP and OXT have been reported to have similar effects on anxiety and social behavior in some species (20, 36), the present results support the hypothesis that they have unique, behaviorally specific programing effects on offspring.

### F1 Maternal OXT and AVP Prevent Increased Anxiety in CSS F2 Offspring

Augmented self-grooming during open-field testing indicates novelty-induced anxiety where juvenile CSS F2 females increased self-grooming when placed in a novel environment. In contrast to the nanopeptide-specific differences in burying behavior, both AVP and OXT treatments prevented the CSS-induced increase in self-grooming during open-field testing. This may suggest context- or behavior-specific effects of AVP and OXT. The lack of effect on general locomotor parameters during open-field testing indicates that the behavioral effects were not mediated by changes in activity levels, consistent with all previous studies of the CSS model.

### F1 Maternal AVP Increases Social Contact in CSS F2 Offspring

In direct support of our previous work on CSS F2 female juveniles, CSS decreased social interaction. Individual group comparisons indicate that AVP, but not OXT, treatment of F1 dams has protective effects on F2 social behavior. This protective effect may be specific to investigatory activities since treatment effects of CSS on F2 allogrooming were similar. AVP is an established mediator of social recognition (37), which may explain its effectiveness in ameliorating the social stress-induced deficits in social interaction through changes in investigatory behaviors, but not allogrooming. In maternal rats, AVP is a key mediator of maternal care, maternal aggression, maternal memory, and self-grooming (38–44), and chronic central infusion of AVP enhances maternal care in CSS-exposed F0 dams (19). The preventative effects of AVP on F2 juveniles indicate that it may be an effective treatment for both depressed maternal care and the adverse effects of deficient care on offspring. Reinforcing the specificity findings in the burying and self-grooming data, AVP and OXT have differential preventative effects on the negative impact of CSS on the F2 social behavior, with AVP having protective effects on overall social contact, yet no effect of either peptide on the CSS-induced decrease in allogrooming.

Studies in voles suggest that that chronic OXT may have adverse long-term effects on social behavior (22), and the present data could represent a similar phenomenon that is transmitted to offspring through changes in maternal care. The lack of preventative effects of F1 dam OXT on social behavior could be related to observations of elevated plasma OXT in CSS F2 juvenile females, which exhibit social deficits (5), and the present findings support related work in humans and rodents on OXT-associated disruptions in social functioning (45–47). Female rodents exposed to different social stressors exhibit long-lasting changes in the OXT system (48, 49). IN OXT is unable to ameliorate stress-induced deficits in mouse social behavior, and OXT decreased social interaction in a control population (49). It is postulated that exogenous OXT treatment of CSS-exposed F2 females may not be effective and could impair social behavior in mothers or their offspring due to context dependent adverse effects (38, 49) on F1 dam maternal care during early lactation when maternal anxiety is elevated (4). Beneficial effects of OXT on social behavior may require more positive social interactions where OXT can enhance the salience of these encounters (50–53).

In addition to the current juvenile social testing paradigm, it is possible that CSS could negatively impact several other forms of social interaction at later life history stages, such as mating, alloparental care (48, 54), parental care (55), and aggression (56, 57) in the F2 generation. The reported changes in juvenile behavior, particularly the decrease in allogrooming, may be predictive of future deficiencies in social bonding and mating behaviors. One possible mechanism supported by the allogrooming data is that depressed F1 maternal care mediates decreased F2 juvenile allogrooming, leading to general deficits in social functioning in the F2 offspring at multiple life history stages. In support of this hypothesis, similarly treated CSS F2 dams exhibit deficits in maternal care (manuscript in review), and additional CSS F2 investigations of mating, alloparental, and parental behavior are warranted.

### Effects of F1 OXT and AVP on F2 Offspring Behavior through Maternal Care

The hypothesis that the effects of chronic F1 IN OXT and AVP treatment on F2 behavior are mediated through changes in F1 maternal care is supported by a wealth of literature on their roles in the establishment and expression of maternal care (20, 58). OXT’s role in maternal behavior has been studied for almost 40 years (59), and there is growing evidence for its role in pathologies that adversely impact parental care. Changes in OXT and dopamine mediate the adverse effects of parental deprivation on the parental care of vole offspring (60), and OXT has been clinically implicated in depression and anxiety (46, 61–63). AVP is also a critical mediator of maternal behavior (38, 64, 65) and the rationale for the current IN AVP treatment was directly supported by increased maternal care in CSS-exposed dams following central AVP infusion (19). Recent clinical evidence supports a key role for AVP V1a receptors in maternal social cognition (66), and animal studies indicate that this role of the V1a receptor may be mediated through hypothalamic nuclei (67). Considering that the F2 females were not treated themselves, there are several possible mechanisms for the effects of the treatments, including changes in F1 maternal care and effects on neuropeptide, inflammatory, or nutritional factors in milk. While IFNγ levels were not correlated with F2 behavior, F1 dam AVP and OXT treatments may have altered IFNγ in milk and F2 offspring and induced immune-mediated neurodevelopmental effects, which mediated the behavioral changes. Key questions to address in exploring AVP- and OXT-induced changes in F1 maternal care are how do both acute and chronic treatments affect F1 maternal care and what are the behavioral effects on F2 offspring. Cross fostering and artificial grooming manipulations may be particularly valuable in these efforts, and changes occurring during early lactation are likely to be critical. Ongoing studies of the CSS model will explore the role of maternal care in the effects of IN AVP and OXT on offspring behavior and immune function.

## Conclusion

Taken together, these results support the hypothesis that maternal AVP and OXT treatment have context- and behavior-specific effects on peripheral IFNγ levels and perseverative, anxiety, and social behaviors in female offspring of early-life social stress-exposed dams. Both maternal AVP and OXT are effective at preventing social stress-induced increases in self-directed measures of anxiety, and AVP is particularly effective at preventing impairments in overall social contact. OXT is specifically effective at preventing repetitive/perseverative behaviors, yet is ineffective at preventing deficits in overall social behavior. Neither treatment was effective in improving allogrooming, which may be suggestive of future impairments in social bonding, mating, and maternal care. A lack of significant IFNγ-behavior correlations suggests that the behavioral effects are not directly mediated by IFNγ and could be mediated by neurodevelopmental effects of this immune factor. The IFNγ data suggest a modulation in immune functioning, which could be relevant to the etiology and pathology of a vast array of stress-associated disorders not explored in the present investigation (68, 69). As the work from the Bales lab has revealed adverse long-term effects of chronic OXT treatment, the present study has revealed that chronic maternal AVP and OXT treatments can have potent effects on the offspring. Studies involving manipulations of potent behavioral mediators need to consider both long-term effects in the treated animal, as well as effects on offspring and future generations. Given the preventative effects of maternal AVP on anxiety and social behavior deficits and the relative lack of data on this neurohormone compared to studies of OXT, increased targeting of AVP for the prevention and treatment of perseverative-, anxiety-, and social behavior-associated disorders may be productive. While the clinical physiological benefits of OXT in the peripartum period are established, potential maternal and offspring effects on immune function and behavior merit further study.

## Ethics Statement

This study was carried out in accordance with the recommendations of the guidelines of the Committee on the Care and Use of Laboratory Animals, National Research Council. The protocol was approved by the Tufts University Institutional Animal Care and Use Committee.

## Author Contributions

CM was involved in the design of the experiment and assisted with data interpretation and completion of the manuscript. AH-N assisted with protocol design, data collection and analysis, and completion of the manuscript. AF assisted with data collection, interpretation, and completion of the manuscript. GB assisted with methodological design, data collection, and interpretation of the results. KG and FE assisted with data collection, interpretation of results, and manuscript completion. FP assisted with interpretation of results and manuscript completion and revision. BN designed and supervised the study, assisted with data collection, organized and integrated the data, and wrote and revised the manuscript.

## Conflict of Interest Statement

The authors declare that the research was conducted in the absence of any commercial or financial relationships that could be construed as a potential conflict of interest.
